# Randomised, open-label, phase II study of gemcitabine with and without IMM-101 for advanced pancreatic cancer

**DOI:** 10.1038/bjc.2016.271

**Published:** 2016-09-06

**Authors:** Angus G Dalgleish, Justin Stebbing, Douglas JA Adamson, Seema Safia Arif, Paolo Bidoli, David Chang, Sue Cheeseman, Robert Diaz-Beveridge, Carlos Fernandez-Martos, Rob Glynne-Jones, Cristina Granetto, Bartomeu Massuti, Karen McAdam, Raymond McDermott, Andrés J Muñoz Martín, Demetris Papamichael, Roberto Pazo-Cid, Jose M Vieitez, Alberto Zaniboni, Kevin J Carroll, Shama Wagle, Andrew Gaya, Satvinder S Mudan

**Affiliations:** 1 Cancer Vaccine Institute, St George's University of London, London, UK; 2 Department of Oncology, Imperial College, Hammersmith Hospital, London, UK; 3 Department of Oncology, Ninewells Hospital, Dundee, UK; 4 Velindre Cancer Centre, Cardiff, UK; 5 Department of Oncology, Azienda Ospedaliera San Gerardo, Monza, Italy; 6 Department of General Surgery, Royal Blackburn Hospital, Blackburn, UK; 7 Department of Oncology, Bradford Teaching Hospitals NHS Foundation Trust, Bradford, UK; 8 Médico Adjunto de Oncología Médica, Hospital La Fe de Valencia, Valencia, Spain; 9 Instituto Valenciano de Oncologia, Valencia, Spain; 10 Mount Vernon Cancer Centre, Northwood, UK; 11 Medical Oncology, Azienda Ospedaliera Santa Croce e Carle, Cuneo, Italy; 12 Ensayos Clínicos Oncología, Hospital General Universitario de Alicante, Alicante, Spain; 13 Oncology Department, Peterborough and Stamford Hospitals NHS Trust, Peterborough, UK; 14 Medical Oncology, St Vincent's University Hospital and The Adelaide and Meath Hospital, Dublin, Republic of Ireland; 15 Gastrointestinal Cancer Unit, Hospital General Universitario Gregorio Marañón, Madrid, Spain; 16 Department of Medical Oncology, Bank of Cyprus Oncology Centre, Nicosia, Cyprus; 17 Servicio de Oncología Médica, Hospital Miguel Servet, Zaragoza, Spain; 18 Area and Neuroendocrine Tumors Gastrointestinal Medical Oncology, Hospital Central de Asturias, Asturias, Spain; 19 Oncology Department, Fondazione Poliambulanza, Brescia, Italy; 20 TranScrip Partners LLP, Reading, UK; 21 Clinical Oncology, Guy's & St Thomas' Hospitals NHS Trust, London, UK; 22 St George's University of London, Imperial College, London and The Royal Marsden Hospital, London, UK

**Keywords:** pancreatic cancer, *Mycobacterium obuense*, phase II, advanced pancreatic ductal adenocarcinoma, immunotherapy, gemcitabine, IMM-101, immunomodulator

## Abstract

**Background:**

Immune Modulation and Gemcitabine Evaluation-1, a randomised, open-label, phase II, first-line, proof of concept study (NCT01303172), explored safety and tolerability of IMM-101 (heat-killed *Mycobacterium obuense*; NCTC 13365) with gemcitabine (GEM) in advanced pancreatic ductal adenocarcinoma.

**Methods:**

Patients were randomised (2 : 1) to IMM-101 (10 mg ml^−l^ intradermally)+GEM (1000 mg m^−2^ intravenously; *n*=75), or GEM alone (*n*=35). Safety was assessed on frequency and incidence of adverse events (AEs). Overall survival (OS), progression-free survival (PFS) and overall response rate (ORR) were collected.

**Results:**

IMM-101 was well tolerated with a similar rate of AE and serious adverse event reporting in both groups after allowance for exposure. Median OS in the intent-to-treat population was 6.7 months for IMM-101+GEM v 5.6 months for GEM; while not significant, the hazard ratio (HR) numerically favoured IMM-101+GEM (HR, 0.68 (95% CI, 0.44–1.04, *P*=0.074). In a pre-defined metastatic subgroup (84%), OS was significantly improved from 4.4 to 7.0 months in favour of IMM-101+GEM (HR, 0.54, 95% CI 0.33–0.87, *P*=0.01).

**Conclusions:**

IMM-101 with GEM was as safe and well tolerated as GEM alone, and there was a suggestion of a beneficial effect on survival in patients with metastatic disease. This warrants further evaluation in an adequately powered confirmatory study.

Only 18% of patients with advanced pancreatic ductal adenocarcinoma (PDAC) remain alive at 1 year, and 4% at 5 years (Hidalgo *et al*, 2015). Survival for metastatic disease is more dismal. Real-world studies report the overall median survival from diagnosis to be 4.6 months; in patients with metastatic cancer the median survival ranges between 2.8 and 5.7 months (Carrato *et al*, 2015).

When IMAGE-1 was set up, gemcitabine (GEM) was the standard of care for advanced PDAC (Burris *et al*, 1997; Network; Seufferlein *et al*, 2012) and at that time it was widely used as the comparator arm in clinical trials for this disease. Over the past 5 years, FOLFIRINOX, as well as GEM+nab-paclitaxel (Abraxane), have entered the clinical arena, mainly for patients with a good performance status because they increase toxicity significantly (Conroy *et al*, 2011; Von Hoff *et al*, 2013). The combination of nab-paclitaxel+GEM demonstrated clinical benefit in the first-line treatment of pancreatic cancer (Von Hoff *et al*, 2013) and was subsequently approved for the first-line treatment of metastatic adenocarcinoma of the pancreas. However, its use in clinical practice has experienced setbacks in Europe, for instance, in 2015, NICE (the National Institute for Health and Care Excellence) did not approve the use of nab-paclitaxel in combination with GEM, but it maintained the recommendation for GEM as the first-line treatment of advanced pancreatic cancer (NICE, 2001; NICE, 2015). The lack of reimbursement for nab-paclitaxel also limits use of this combination in the Benelux countries, Ireland and Eastern Europe. Despite the recent advances of FOLFIRINOX and nab-paclitaxel+GEM in the treatment of pancreatic cancer, the majority of pancreatic cancer patients (76%) do not receive either of these regimens as first-line treatment (Braiteh *et al*, 2016). Therefore, an unmet need remains for therapies that confer meaningful survival advantages without additional toxicity.

Immunotherapy is effective in treating many cancers (Tempero *et al*, 2012), although success in PDAC is limited (Gunturu *et al*, 2013; Pico de Coana *et al*, 2015). Extended survival after second-line treatment with the therapeutic vaccine GVAX and CRS-207 (live-attenuated *Listeria monocytogenes*) has been reported after the treatment with low-dose cyclophosphamide (Le *et al*, 2015).

IMM-101 is a systemic immune modulator containing heat-killed *Mycobacterium obuense* (NCTC 13365). Results from *in vivo* and *ex vivo* non-clinical studies suggest that IMM-101 modulates the innate and adaptive immune systems, in response to cancer. IMM-101 acts on cells of the innate immune system, such as γδ T-cells, granulocytes, and antigen-presenting cells, by interaction with a number of receptors (PAMPs-PRR; Fowler *et al*, 2011) (Bazzi *et al*, 2015). Activation of these cells is known to have a cytotoxic effect against tumours. Furthermore, it is proposed that IMM-101 restores Type-1 response, influences cytotoxic cell immune function and may downregulate Type 2 response. This is of significance because pancreatic cancer has been associated with a T_h_2 bias (Wormann *et al*, 2014).

In a phase I clinical study, IMM-101 was safe and well tolerated at the three escalating doses used in patients with melanoma (Stebbing *et al*, 2012). Therefore, IMAGE-1 was designed as a proof of concept (POC), phase II study primarily to explore the safety and tolerability of IMM-101 in combination with GEM *vs* GEM alone as first-line treatment in advanced PDAC. In addition, the study would provide some insight into the potential effects of treatment on the clinical signs and symptoms of disease including overall survival (OS), progressive-free survival (PFS) and overall response rate (ORR).

## Materials and Methods

### Study design and patients

This open-label, phase II trial was conducted at 20 institutions in 5 countries (Cyprus, Ireland, Italy, Spain, UK). Eligible patients were age ⩾18 years, had confirmed inoperable PDAC (with or without metastatic disease), measurable lesions at ⩾1 site not previously irradiated, and WHO performance status (PS) 0–2. Other inclusion criteria included serum albumin ⩾26 g l^−6^, C-reactive protein (CRP) ⩽70 mg l^−1^, and life expectancy >3 months from randomisation. Exclusion criteria included prior PDAC chemotherapy, radiotherapy within 6 weeks of screening and chronic use of corticosteroids within 2 weeks of first study drug. The study was undertaken in compliance with the Declaration of Helsinki Principles and applicable local–regional regulations. The study protocol, the patient information leaflet and informed consent form were reviewed and approved by an Independent Ethics Committee/Institutional Review Board. All patients provided written informed consent. Patients were randomly assigned in a 2 : 1 ratio to receive IMM-101+GEM or GEM alone by Interactive Response Technology. Randomisation was stratified according to disease extent and WHO PS, by computer generated block randomisation methods.

### Procedures

In both groups, GEM was administered intravenously at 1000 mg m^−^^2^ over 30 min weekly for 3 weeks out of 4, with dose reductions allowed for toxicity. IMM-101 (0.1 ml of 10 mg ml^−1^ suspension) was administered by intradermal injection into the skin overlying the deltoid muscle with the arm alternated for each dose. This dose of IMM-101 was previously shown to be safe and well tolerated in patients with melanoma (Stebbing *et al*, 2012). Dosing delays or half doses were allowed if skin reaction was unacceptable. IMM-101 was administered every 2 weeks for three doses followed by 4 weeks rest, then every 2 weeks for a further three doses. Subsequent doses were administered every 4 weeks; the first IMM-101 dose was administered 2 weeks before the first dose of GEM. Upon disease progression or toxicity to GEM, second-line chemotherapy of the investigator's choice was allowed.

Maximum treatment duration was 12 cycles. All patients who completed the study (from both treatment groups) were able to enter a long-term follow-up study in which all would receive IMM-101.

### Assessments

Adverse events (AEs) were reported at each visit according to National Cancer Institute Common Terminology Criteria for Adverse Events version 4.0 (DCTD, NHI, DHHS, Bethesda, MD, USA; http://cstep.cancer.gov/reporting/ctc.html). To assess the impact on toxicity of the time on study treatment, the rates of patients per month on study reporting at least one AE or at least one serious AE (SAE) were calculated. Injection site reactions to IMM-101 were recorded at each visit and included assessments of pain, induration, wet drainage, erythema, and tenderness as well as any impact on daily activities. Tumour response was determined by investigator assessment at baseline, weeks 13, 25, 37, and 48, and as clinically indicated according to RECIST v1.1 (Brussels, Belgium; http://www.eortc.be/recist). Complete (CR) or partial responses (PR), and stable disease beyond 3 months were confirmed by a second radiologist.

### Statistical analysis

As an exploratory, phase II, POC study with safety and tolerability assessment as the primary endpoint, the trial was not formally sized to test a specific efficacy hypothesis. A target of 90 patients (on a 2 : 1 allocation basis) was considered feasible and sufficient to address the primary endpoint. This number of patients is broadly in line with the size of other randomised, phase II trials in oncology, and was considered sufficient to provide insight into the potential efficacy of IMM-101 on additional endpoints including OS, PFS, and ORR.

Safety assessment was based on frequency and incidence of AEs using the safety analysis set of all patients who received study drug. OS and PFS outcomes were displayed as Kaplan–Meier curves for the intent-to-treat (ITT) analysis set and for the metastatic and locally advanced subgroups (only a selection of Kaplan-Meier curves will be displayed in this paper). Median survival estimates as well as 95% confidence intervals (CI) were reported for each group. OS and PFS differences were tested by two-sided log-rank tests. Cox proportional hazard (PH) regression models were used to estimate hazard ratios (HR) with 95% CI. Data were censored if patients remained alive (OS), or had no recorded progression (PFS) at the time of analysis, or were lost to follow-up. Survival times were calculated from the date of randomisation until death. PFS was defined as the interval between randomisation and radiological and/or clinical progression or death. ORR was defined as a complete or partial response. Disease stabilisation included those patients with a response and also stable disease ⩾3 months.

To assess the potential influence of baseline characteristics on survival and PFS outcomes, an exploratory multivariate stepwise analysis (Cox PH regression model) was conducted for factors reported as prognostic for survival in PDAC (carbohydrate antigen 19.9 (CA19.9), carcinoembryonic antigen (CEA), lactate dehydrogenase (LDH), CRP, neutrophil-lymphocyte ratio (NLR), total bilirubin, age, and PS) (Bilici, 2014; Haas *et al*, 2013) alongside treatment group for the ITT and metastatic subgroup.

## Results

### Patients and treatment

**Figure 1:**
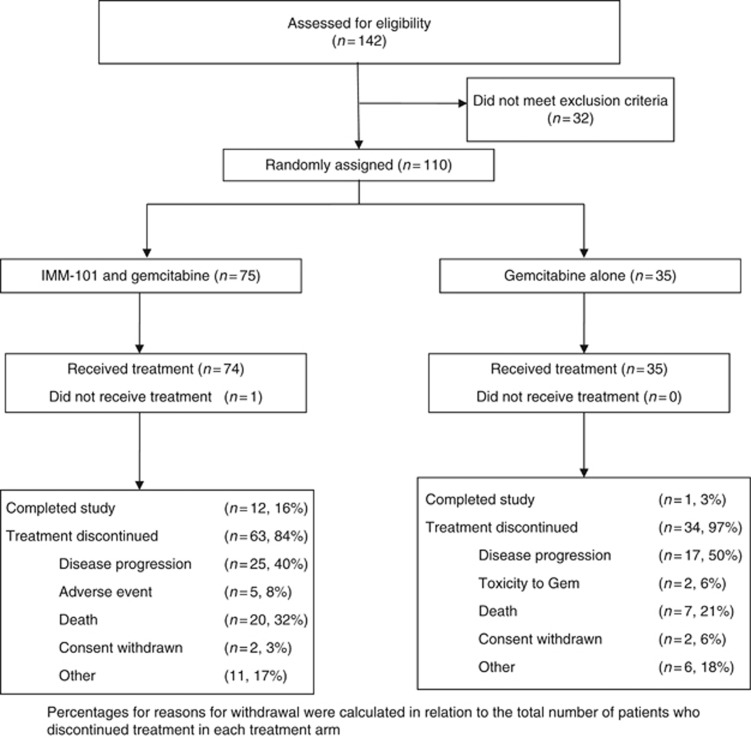
CONSORT diagram.

Between July 2011 and August 2013, 110 patients were enrolled and randomised (Figure 1). The ITT analysis included all randomised patients. Five patients in the IMM-101+GEM group and one patient in the GEM group remained alive and were censored for OS at the last point of follow up. A further six patients in the IMM-101+GEM group and two patients in the GEM group were lost to follow-up and censored at the last allowed date of follow-up. Demographic and baseline characteristics were mostly balanced between the treatment groups, but with some differences observed for age distribution, gender, PS, time since diagnosis, CA19.9 and NLR (Table 1). 84% of patients had metastatic disease on enrolment.

### Safety and exposure

The Safety population was the same as the ITT population, except that one patient was excluded from the IMM-101+GEM group, having been withdrawn before study drug administration.

Median time on study was 4.83 months (range 0.2–12.0) for IMM-101+GEM, and 2.79 months (0.5–10.9) for GEM. The total time on study was 414 months for the IMM-101+GEM group and 133 months for the GEM group. Median duration of exposure to GEM was longer for IMM-101+GEM compared to GEM (78 and 59 days, respectively). To assess the impact on toxicity of the longer time on study observed for the IMM-101+GEM group, the rates of patients per month on study reporting at least one AE or at least one SAE were also calculated and are reported here.

Seventy three (99%) patients reported at least one AE in the IMM-101+GEM group compared with 35 (100%) patients in the GEM group. The corresponding rate of patients reporting at least one AE per month on study for IMM-101+GEM *vs* GEM was 0.18 *vs* 0.26. Pyrexia occurred with the greatest difference in incidence between IMM-101+GEM *vs* GEM (28.4% v 8.6%), with all cases in the IMM-101+GEM group being grade 1 (majority) or grade 2. Grade 3 and higher AEs occurred in 57 (77%) patients in the IMM-101+GEM group and 26 (74%) patients in the GEM group. All grade 3 and higher AEs with an incidence of ⩾5% in either group are shown in Table 2.

Thirty six patients reported at least one SAE in the IMM-101+GEM group compared to 10 patients in the GEM group. The corresponding rate of patients reporting at least one SAE per month on study for IMM-101+GEM *vs* GEM was 0.09 *vs* 0.08. SAEs which occurred in ⩾5% of IMM-101+GEM treated patients were biliary sepsis, abdominal pain and pyrexia (each occurring in 5% of patients) and disease progression in the GEM group (6%). The incidence of individual SAEs was low and no trend could be observed.

Fatal (grade 5) AEs were reported in 19% of the IMM-101+GEM group *vs* 14% in GEM, although none were considered related to study drug.

IMM-101 injection site reactions were almost all mild or moderate with isolated severe reactions in 4% of patients who all subsequently continued treatment. Only 2 patients (3%) required a reduction to half dose as a result of local reactions, and both completed the study. The worst impact of IMM-101 injection on patients' daily activities related to 8% reporting a moderate impact which resolved during their time on study.

Treatment-related AEs leading to withdrawal from study were reported in 5% of the IMM-101+GEM group (all but 1 event related to GEM and two also related to IMM-101) *vs* none in GEM.

### Efficacy

**Figure 2:**
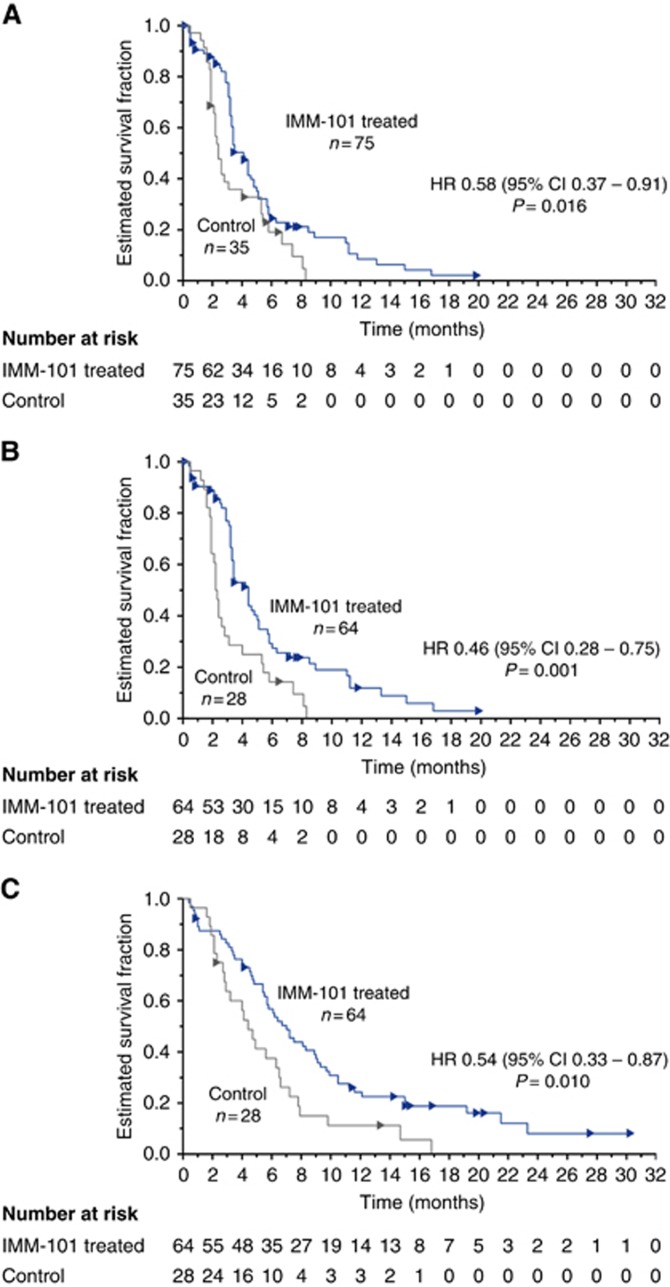
Progression-free survival ITT Analysis Set (**A**) and ITT Metastatic Subgroup (**B**). Overall survival Metastatic Subgroup (**C**). Arrows denote censored events. IMM-101 treated: IMM-101+GEM; Control: GEM alone.

Survival analysis of the ITT group included deaths in 85% of the IMM-101+GEM group and 91% of the GEM group, with median follow-up of 6.7 months (range 0.4–30.3) and 4.9 months (0.5–16.8), respectively. Table 3 and Figure 2 show the results for median OS and PFS.

Median OS in the ITT population was 6.7 months for IMM-101+GEM v 5.6 months for GEM (HR, 0.68; 95% CI, 0.44–1.04, *P*=0.074); the difference was not formally statistically significant. 28% of patients in the IMM-101+GEM group and 34% in the GEM group took second-line therapy; 15 patients from the IMM-101+GEM group continued to receive IMM-101.

Analysis of the predefined metastatic subgroup (*n*=92) showed a difference in survival between the treatment groups with median survival for IMM-101+GEM of 7.0 *vs* 4.4 months for GEM (HR, 0.54; 95% CI 0.33–0.87, *P*=0.01). The smaller subgroup of patients with locally advanced disease (*n*=18) showed a lower median survival for IMM-101+GEM of 6.7 months *vs* 9.2 months for GEM (HR, 3.81; 95% CI 1.03–14.05, *P*=0.032). Results are difficult to interpret in the latter subgroup because of the low number of patients.

The planned survival analysis based on PS (0–1 *vs* 2) was not performed because the PS2 subgroup from the GEM arm contained only thre patients.

Results for PFS reflected those for OS. In the ITT population, median PFS was 4.1 months for IMM-101+GEM *vs* 2.4 months for GEM (HR, 0.58; 95% CI 0.37–0.91; *P*=0.016); the difference was statistically significant. For the metastatic subgroup, median PFS was 4.4 months for IMM-101+GEM *vs* 2.3 months for GEM (HR 0.46; 95% CI 0.28–0.75; *P*=0.001). The small subgroup with locally advanced disease had median PFS of 3.4 months for IMM-101+GEM *vs* 5.3 months for GEM (HR 2.38; 95% CI 0.65–8.78; *P*=0.177).

The exploratory multivariate analysis of PFS and OS outcomes indicated that baseline CA19.9, CEA, CRP, NLR and randomised treatment were prognostic for PFS outcome in the ITT population and metastatic subgroup and also for OS outcome in the metastatic subgroup. For OS in the ITT population, randomised treatment fell marginally short of the multivariate stepwise inclusion criteria. Overall, these exploratory analyses confirmed that the differences seen for PFS and OS between IMM-101+GEM v GEM were not attributable to important prognostic factors and any associated chance baseline imbalances. An exploratory multivariate analysis was not performed on the subgroup with locally advanced disease because the number of patients was too small.

The ORR of 10.7% was numerically higher for IMM-101+GEM (95% CI 4.7–19.9) v 2.9% for GEM (95% CI 0.1–14.9; *P*=0.164). The best overall response was a PR (i.e., there were no complete responders).

The disease stabilisation rate was 44% (95% CI 32.5–55.9) for IMM-101+GEM and 34.3% (95% CI 19.1–52.2, *P*=0.334) for GEM.

## Discussion

IMAGE-1 is the first, randomised, phase II, POC study to explore the safety and tolerability of IMM-101 in combination with GEM *vs* GEM alone as first-line treatment in advanced PDAC. IMM-101+GEM was as well tolerated as GEM alone: the rates of patients per month on study reporting at least one AE or at least one SAE were similar between treatment groups.

Pyrexia is typical post-vaccination, and was more frequent in the IMM-101+GEM group, with all cases classified as Grade 1 or 2. Injection–site reactions are a predictable reaction to mycobacterial antigens and were well tolerated by patients.

Grade 3 and higher AEs occurred at a similar incidence between treatment arms. No treatment-related deaths occurred.

This POC study was not formally sized to test a specific efficacy hypothesis, nonetheless, it has provided important insights into the potential for efficacy improvements with the use of IMM-101 in PDAC. For the overall ITT population, the median OS was similar between treatment groups while PFS was greater in the IMM-101+GEM group.

The pre-planned subgroup analysis of patients with locally advanced disease contained only 16% of the ITT population; *n*=11 for IMM-101+GEM and *n*=7 for GEM with just 11 and 6 deaths, respectively. Consequently, analyses in locally advanced patients lack robustness and are associated with wide CIs. In contrast, the pre-planned subgroup analysis of patients with metastatic disease contained 84% of the ITT population, and therefore, provide more reliable evidence regarding the possible effect of IMM-101+ GEM v GEM in this population. HRs and median OS and PFS were higher in the IMM-101+GEM group, and associated CIs were narrower so increasing the confidence of the possibility of a true survival benefit in the IMM-101+GEM group.

In a study of this size, with 2 : 1 randomisation, there is the chance that any apparent treatment benefit may be due to an imbalance in baseline characteristics, and some degree of imbalance for certain factors was noted (Table 1). However, a multivariate analysis based on factors reported as prognostic for survival in the literature (Bilici, 2014; Haas *et al*, 2013), and for which data were collected, showed, overall, that the differences seen for PFS and OS between the IMM-101+GEM group *vs* GEM were not attributable to important prognostic factors and any associated chance baseline imbalances. Missing data from some patients (maximum of 15% in IMM-101+GEM group and 14% in the GEM group) may have influenced results. Data were not collected on the site of primary tumour (head/body) which has been shown to be prognostic in some studies, although without a clear consensus (Bilici, 2014).

The median survival times for the GEM group were relatively low compared with some published information for pancreatic cancer patients receiving GEM monotherapy. The younger age of the study population in recently published phase III studies (58% <age 65 years in MPACT (Von Hoff *et al*, 2013) and 71% ⩽age 65 years in FOLFIRINOX trial (Conroy *et al*, 2011)) may have contributed to improved OS in those studies, although age was not shown to be prognostic for survival in IMAGE-1 which had 60% of patients aged over 65 years. Published median OS for patients receiving GEM monotherapy shows considerable variation from 4.9 months (Poplin *et al*, 2009) to 8.3 months (Colucci *et al*, 2010) (for predominantly metastatic populations). In a systematic review, median survival for patients with pancreatic cancer who underwent interventions (including chemotherapy, radiotherapy or surgery) ranged from 2–8.1 months (Carrato *et al*, 2015). In IMAGE-1, the relatively long time from diagnosis (Table 1) with eligibility not restricted to newly diagnosed patients, may have reduced median OS in both arms.

Upon disease progression or toxicity to GEM, the protocol allowed treatment changes to be made on study, rather than only after withdrawal. This facilitated data collection and allowed patients from the IMM-101+GEM group to continue to receive IMM-101. The use of second-line anticancer therapy was balanced between treatment groups with 28% of patients in the IMM-101+GEM group and 34% in the GEM group; this had no bearing on the ITT analysis.

In this study, IMM-101 in combination with GEM was as safe and well tolerated as GEM alone in patients with advanced PDAC, and there was a suggestion of a beneficial effect on survival in patients with metastatic disease. This supports further evaluation of IMM-101 in an adequately powered confirmatory study. Moreover, ongoing analyses to identify potentially predictive markers of response will guide the design of this new study.

Currently there are 150 studies listed on https://clinicaltrials.gov/ that are investigating the combination of GEM with nab-paclitaxel in different combination regimens for pancreatic cancer. Where reported, the study populations are significantly younger and clinically fitter than in IMAGE-1.

There are also several studies evaluating combinations with FOLFIRINOX. While promising activity has been seen as judged by response rate (Nywening *et al*, 2016), the toxicity of FOLFIRINOX-based regimens precludes treatment of the majority of patients with advanced pancreatic cancer.

The clinical crux is that GEM still represents the clinical option for patients with poorer performance status in this disease. In addition, single agent GEM still continues to be the standard of care for patients who are not fit for FOLFIRINOX in economically restricted health care systems globally. Therefore, our intention is to plan a large, adequately powered, phase III study of IMM-101 in combination with GEM for the first-line treatment of patients with metastatic pancreatic cancer. The patient population that is eligible for this study will be defined carefully to ensure that only those patients who are not suitable for treatment with GEM+nab-paclitaxel or FOLFIRINOX are enroled.

We do acknowledge the need to investigate IMM-101 in combination with standard of care in first-line treatment of metastatic pancreatic cancer, and also in second-line treatment of metastatic pancreatic cancer in combination with MM-398, a nanoliposomal encapsulation of irinotecan,+5-fluorouracil and folinic acid (Wang-Gillam *et al*, 2016). Therefore, a follow-up phase I/IIa trial to evaluate the safety, tolerability and activity of IMM-101 in combination with different chemotherapy regimens in pancreatic cancer is currently being instigated.

## Figures and Tables

**Table 1:** Baseline characteristics of all enrolled patients

	**IMM-101+GEM (*****n*****=75)**	**GEM (*****n*****=35)**
**Age, years**		
Median	68	66
Range	45–88	53–83
Distribution, no (%)		
⩽65	28 (37)	16 (46)
>65	47 (63)	19 (54)
**Sex, no (%)**		
Female	37 (49)	14 (40)
Male	38 (51)	21 (60)
**Race, no (%)**		
White	74 (99)	33 (94)
Asian	1 (1)	0
Other	0	1 (3)
Unknown	0	1 (3)
**ECOG performance status (PS), no (%)**		
0–1	62 (83)	32 (91)
2	13 (17)	3 (9)
**Time since first diagnosis, months**		
Median	1.22	0.76
Range	0.1–6.9	0.1–3.9
**Extent of disease, no (%)**		
Locally advanced	11 (15)	7 (20)
Metastatic	64 (85)	28 (80)
**CA19.9, KU l^−1^**		
Median	485.8	2747
Range	0.6–455, 480	0.1–100 000
Distribution, no (%)		
⩽1000	38 (58)	11 (32)
>1000	27 (42)	23 (68)
**CEA, μg l^−1^**		
Median	10	9.3
Range	0.7–679.9	2.0–681.0
Distribution, no (%)		
⩽10	33 (51)	17 (55)
>10	32 (49)	14 (45)
**LDH, U l^−1^**		
Median	198.5	221
Range	118–2101	145–643
Distribution, no (%)		
⩽250	42 (66)	18 (60)
>250	22 (34)	12 (40)
**CRP, mg l^−1^**		
Median	10	11.9
Range	0.8–330.0	0.6–76.8
Distribution, no (%)		
⩽10	37 (51)	16 (46)
>10	35 (49)	19 (54)
**Total bilirubin, mg dl^−1^**		
Median	0.6	0.68
Range	0.24–4.40	0.24–2.04
Distribution, no (%)		
⩽1	52 (69)	21 (62)
>1	23 (31)	13 (38)
**NLR**		
Median	3.42	3.91
Range	1.24–1518.52	0.53–9.11
Distribution, no (%)		
⩽5	55 (73)	28 (80)
>5	20 (27)	7 (20)

Abbreviations: CA19.9=carbohydrate antigen 19.9; CEA=carcinoembryonic antigen; CRP=C-reactive protein; ECOG=Eastern Cooperative Oncology Group; GEM=gemcitabine; LDH=lactate dehydrogenase, NLR=neutrophil-lymphocyte ratio.

**Table 2:** Grade 3 and higher adverse events occurring in at least 5% patients in either group

	**Number of patients (%)**		
**NCI CTC adverse events**	**IMM-101+GEM (*****n*****=74)**	**GEM (*****n*****=35)**	**Difference in Incidence Rates (IMM-101+GEM–GEM)**
Asthenia	8 (11%)	1 (3%)	8%
Abdominal pain	6 (8%)	1 (3%)	5%
Vomiting	4 (5%)	0	5%
Anaemia	6 (8%)	1 (3%)	5%
Biliary sepsis	4 (5%)	0	5%
Bile duct obstruction	4 (5%)	1 (3%)	2%
Neutropenia and/or neutrophil count decreased	13 (18%)	6 (17%)	1%
Leukopenia and/or WBC count decreased	3 (4%)	4 (11%)	−7%
Hypokalaemia and/or blood potassium decreased	0	2 (6%)	−6%
Fatigue	4 (5%)	4 (11%)	−6%
Urinary tract infection	1 (1%)	2 (6%)	−5%
Disease progression	3 (4%)	3 (9%)	−5%
Thrombocytopenia and/or platelet count decreased	5 (7%)	3 (9%)	−2%
ALT increased	3 (4%)	2 (6%)	−2%

Abbreviations: ALT=alanine transaminase; GEM=gemcitabine; NCI CTC=National Cancer Institute Common Terminology Criteria; WBC= white blood cells.

**Table 3:** Overall survival and progression-free survival

	**Median survival, months (95% CI)**			
	**IMM-101+GEM**	**GEM**	**Hazard ratio (95% CI)**	**Log rank*****P*****-value (two sided)**
All patients	6.7 (5.4–7.5) (*n*=75)	5.6 (3.2–7.2) (*n*=35)	0.68 (0.44–1.04)	0.074
Metastatic subgroup	7.0 (5.5–9.0) (*n*=64)	4.4 (2.8–6.5) (*n*=28)	0.54 (0.33–0.87)	0.01
Locally advanced disease subgroup	6.7 (1.4–7.2) (*n*=11)	9.2 (3.5–15.9) (*n*=7)	3.81 (1.03–14.05)	0.032

	**Median progression-free survival, months (95% CI)**			
	**IMM-101+GEM**	**GEM**	**Hazard ratio (95% CI)**	**Log rank*****P*****-value (two sided)**
All patients	4.1 (3.3–4.8) (*n*=75)	2.4 (2.1–4.0) (*n*=35)	0.58 (0.37–0.91)	0.016
Metastatic subgroup	4.4 (3.3–5.1) (*n*=64)	2.3 (1.9–2.8) (*n*=28)	0.46 (0.28–0.75)	0.001
Locally advanced disease subgroup	3.4 (1.4–4.8) (*n*=11)	5.3 (1.9–6.7) (*n*=7)	2.38 (0.65–8.78)	0.177

Abbreviations: CI= confidence interval; GEM= gemcitabine.
